# Regulatory role of miR-142-3p on the functional hepatic cancer stem cell marker CD133

**DOI:** 10.18632/oncotarget.2167

**Published:** 2014-07-05

**Authors:** Stella Chai, Man Tong, Kai Yu Ng, Pak Shing Kwan, Yuen Piu Chan, Tsun Ming Fung, Terence K. Lee, Nathalie Wong, Dan Xie, Yun-Fei Yuan, Xin-Yuan Guan, Stephanie Ma

**Affiliations:** ^1^ Department of Anatomy, The University of Hong Kong, Hong Kong; ^2^ Department of Clinical Oncology, The University of Hong Kong, Hong Kong; ^3^ Departments of Pathology, The University of Hong Kong, Hong Kong; ^4^ State Key Laboratory for Liver Research, The University of Hong Kong, Hong Kong; ^5^ Centre for Cancer Research, Li Ka Shing Faculty of Medicine, The University of Hong Kong, Hong Kong; ^6^ Department of Anatomical and Cellular Pathology, The Chinese University of Hong Kong; ^7^ State Key Laboratory of Oncology in South China, Sun Yat-Sen University Cancer Centre, Guangzhou, China

**Keywords:** CD133, miR-142-3p, tumor-initiating cells

## Abstract

Tumor relapse after therapy typifies hepatocellular carcinoma (HCC) and is believed to be attributable to residual cancer stem cells (CSCs) that survive treatment. We have previously identified a CSC population derived from HCC that is characterized by CD133. Despite our growing knowledge of the importance of this subset of cells in driving HCC, the regulatory mechanism of CD133 is not known. Epigenetic changes are believed to be essential in the control of cancer and stem cells. Here, we report the epigenetic regulation of CD133 by miR-142-3p. The interaction between CD133 and miR-142-3p was identified by in silico prediction and substantiated by luciferase reporter analysis. Expression of CD133 was found to be inversely correlated with miR-142-3p in HCC clinical samples as well as in cell lines. Importantly, lower miR-142-3p expression in HCC was significantly associated with worst survival. Functional studies with miR-142-3p stably transduced in HCC cells demonstrated a diminished ability to self-renew, initiate tumor growth, invade, migrate, induce angiogenesis and resist chemotherapy. Rescue experiments whereby CD133 and miR-142-3p is simultaneously overexpressed compensated the deregulated ability of the cells to confer these features. Thus, miR-142-3p directly targets CD133 to regulate its ability to confer cancer and stem cell-like features in HCC.

## INTRODUCTION

Hepatocellular carcinoma (HCC) is the most commonly diagnosed malignancy of the liver and ranks the fifth most frequently diagnosed cancer in the world. The overall prognosis of HCC is dismal due to the high rate of recurrence and the chemotherapy resistant nature of the tumor. Recently, the cancer stem cell (CSC) model has helped explain why tumor eradication has not been achieved despite advances in treatment. The CSC hypothesis posits that malignant growth arises from a rare subset of cells within a tumor that provide it with unlimited self-renewal and tumorigenic capacity. We and others have previously identified a specific subset of liver CSCs that is marked by their CD133 surface phenotype. CD133^+^ liver CSCs bear features that include the abilities to self-renew, differentiate, resist standard chemotherapy and initiate tumors at limited dilution [1-3]. CD133 is not just a marker of liver CSCs. It was also found to play an important functional role in regulating liver tumorigensis as evident by CD133 shRNA knockdown experiments [4]. Clinically, CD133 expression was also found to have prognostic value in HCC as its presence was associated with worse overall survival and higher recurrence rates. Importantly, our results are consistent with studies by other groups where liver CSCs are also found to be marked by CD133 and that CD133 expression is an important risk factor for overall survival in HCC, further demonstrating the prominence of CD133 in the cancer [5-9]. Findings by us and others now clearly suggest the importance of a CD133 liver CSC subset in driving HCC. However, the underlying molecular mechanism by which CD133 is regulated is still not clear.

Epigenetic changes, including miRNA regulation, promoter methylation and histone modification, are believed to be integral to the behavior of CSCs and their progeny [10]. In particular, there has been increasing evidence in support of a role of miRNA in the regulation of cancer stem cell-like properties in CSCs in recent years [11-12]. Specifically in HCC, although there is increasing work in the study of deregulated miRNAs in various liver CSC subsets as compared with non-CSC differentiated counterparts, for example miR-181 in EpCAM^+^ liver CSCs [13], miR-150 up-regulation in CD133^+^ liver CSCs [14] and our previous work on miR-130b and TP53INP1 interaction in CD133^+^ liver CSCs [3], the role of miRNAs in the direct regulation of CD133 in HCC has not been explored.

Here, we report the epigenetic regulation of the functional liver CSC marker CD133 by miR-142-3p. By in silico prediction analysis, we found the 3'UTR of CD133 to encompass a putative binding region bearing significant complementarity against miR-142-3p. The bona fide interaction between CD133 and miR-142-3p was validated by luciferase reporter assays. Expression of CD133 was found to be inversely correlated with miR-142-3p in a panel of liver cell lines with different CD133 expression levels. Likewise, expression of miR-142-3p was also significantly repressed in HCC clinical samples, as compared with adjacent non-tumor liver tissues. This is inversely correlated with CD133 expression in HCC where we previously reported CD133 to be preferentially expressed in HCC, but is detected at only low or absent levels in non-tumor liver tissues [1, 3]. Lower miR-142-3p expression in HCC was significantly associated with worst disease free survival. miR-142-3p was also found to be preferentially expressed in the CD133^-^ subset isolated from HCC cells PLC8024, Huh7 and SNU182 as compared to its CD133^+^ counterpart. In addition, we also observed an inverse CD133 pattern, at both genomic and proteomic levels, following lentiviral up-regulation of miR-142-3p in CD133-expressing Huh7 and PLC8024 HCC cells. Functional studies in Huh7 and PLC8024 with miR-142-3p stably transduced demonstrated a diminished ability to self-renew, initiate tumor growth, invade, migrate, induce capillary tube formation in endothelial cells and resist standard chemotherapy (cisplatin and 5-fluorouracil). Rescue experiments whereby CD133 is overexpressed in Huh7 with miR-142-3p already stably transduced compensated the deregulated ability of the cells to confer the above stated cancer and stem cell-like features. Taken together, our results show that miR-142-3p plays a critical role in the control of the downstream functional CSC target CD133 in HCC.

## RESULTS

### CD133 is a direct target of miR-142-3p

**Figure 1:**
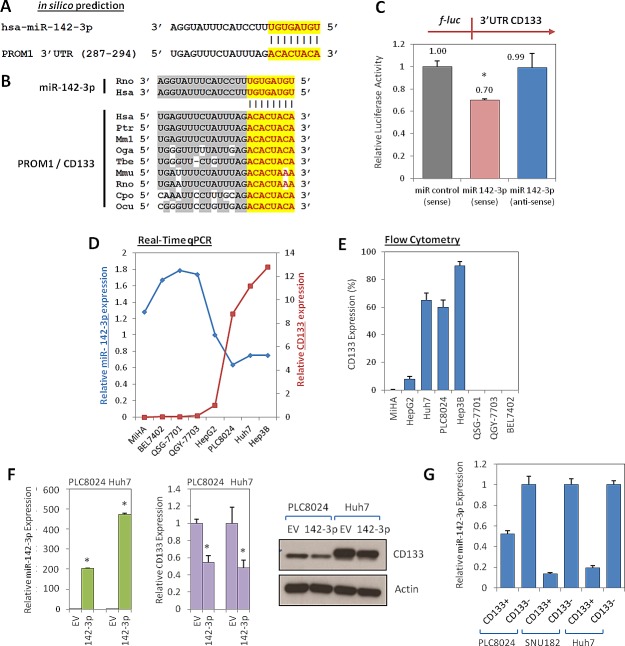
Regulation of CD133 by miR-142-3p (A) In silico prediction identified CD133 to be a target of miR-142-3p. (B) The chimpanzee (ptr: *pan troglodytes*), monkey (mml: *macaca mulatta*), bushbaby (oga; *otolemur garnetti*), treeshrew (tbe: *tupaia belangeri*), mouse (mmu: *mus musculus*), rat (rno: *rattus norvegicus*), guinea pig (cpo: *cavia porcellus*), rabbit (ocu: *oryctolagus cuniculus*) and human (hsa: *homo sapiens*) miR-142-3p and the predicted binding site in the 3'UTR of CD133 in different species were checked for alignment. The positions of the miRNA binding sites corresponded to the location of the GenBank sequence NM_006017. (C) Validation of miR-142-3p binding to the 3'UTR of CD133 by lucifearse reporter assay. Full length 3'UTR of CD133 (full-length) was PCR amplified and then cloned downstream of a firefly luciferase gene. Specificity of the inhibition was determined using anti-sense constructs (3' to 5' orientation). The pRL-TK Renilla luciferase plasmid was co-transfected as a normalization control for firefly luciferase activity. (D) The inverse correlations of CD133 (red line) and miR-142-3p (blue line) expression were determined across a panel of liver cell lines by qPCR. HepG2 was used as the calibrator. (E) Flow cytometry analysis of glycosylated protein CD133 expression in the same panel of liver cell lines. (F) Left panel: Stable overexpression of miR-142-3p in HCC cells PLC8024 and Huh7 by lentiviral transduction was confirmed by qPCR analysis. Middle and right panels: qPCR and Western blot analyses of CD133 expression following stable transduction of miR-142-3p or empty vector (EV) control in HCC cell lines PLC8024 and Huh7. (G) qPCR analysis for miR-142-3p expression in sorted CD133^+^ and CD133^-^ subsets isolated from HCC cells PLC8024, SNU182 and Huh7.

By flow cytometry and immunohistochemistry analyses on both freshly resected and paraffin embedded clinical tissue samples, we [1, 3] and others [6] have previously reported that the liver CSC marker CD133 is represented only in a small subset of the tumor population (~0.5 – 25%) in human HCC specimens. Its expression had prognostic value in HCC as its presence was associated with worse overall survival and higher recurrence rates [3]. In contrast, CD133 expression in normal hepatocytes, biliary epithelial structures in portal area or non-tumor liver tissues adjacent to HCC was significantly lower, if not, completely absent at times [3, 6]. Despite the importance of CD133 in cancer and stem cell biology, limited is known about the regulation of CD133 expression in HCC. Recent studies have implicated miRNAs as prominent factors that contribute to the phenotype of cancer stem cells [10]. Here, we hypothesized whether epigenetic miRNA regulation would also play a role in regulating CD133 in HCC. In an effort to identify human miRNAs that potentially regulate CD133, in silico prediction softwares TargetScan and miRanda were used to screen for common candidate miRNAs that bind to the 3'UTR of CD133. Using this method, only one conserved miRNA (miR-142-3p) was found to have predicted consequential pairing with the 3'UTR of CD133 with a 8mer seed match. Based-pairing complementation found the 3'UTR of CD133 to encompass a putative binding region (position 287-294) bearing significant 8mer complementarity against miR-142-3p (Figure 1A). This 3'UTR element of CD133 and miR-142-3p are extremely conserved among different species, as shown by their identical sequences in chimpanzee, monkey, mouse, rat, guinea pig, rabbit and human orthologs, suggesting a functional role (Figure 1B). To validate whether CD133 is a bona fide target of miR-142-3p, a full-length human CD133 3'UTR fragment was cloned downstream of the *firefly* luciferase reporter gene. Compared with miR-control experiments, luciferase activity was markedly reduced by approximately 30% in the cells co-transfected with miR-142-3p and CD133 3'UTR in the sense direction. As a reflection of specificity, this inhibitory effect was abolished when anti-sense 3'UTR CD133 was used in place of the sense construct (Figure 1C).

Interestingly, the expression of CD133 was also found to be inversely correlated with miR-142-3p in a series of liver cell lines expressing different CD133 expression levels. Liver cell lines with absent or low CD133 expression had relatively higher levels of miR-142-3p (MiHA, BEL7402, QSG-7701, QGY-7703 and HepG2), while, in contrast, liver cell lines with high CD133 levels expressed relatively lower levels of miR-142-3p (PLC8024, Huh7 and Hep3B) (Figure 1D). Glycosylated CD133 at the proteomic level was further analyzed by flow cytometry, where its expression was found to closely match with its genomic level as detected by qPCR (Figure 1E), suggesting the inverse correlation extends to the proteomic level. In addition, we also observed an inverse CD133 pattern, at both genomic and proteomic levels, following lentiviral up-regulation of miR-142-3p in CD133-expressing Huh7 and PLC8024 HCC cells, as compared to empty vector (EV) controls (Figure 1F). miR-142-3p was also found to be preferentially expressed in the CD133^-^ subset isolated from HCC cells PLC8024, SNU182 and Huh7 as compared to its CD133^+^ counterpart (Figure 1G).

### miR-142-3p is frequently under-expressed in HCC and lower expression is significantly associated with worst overall survival

**Figure 2:**
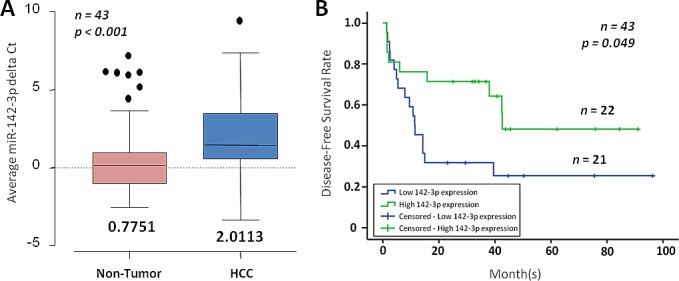
miR-142-3p is frequently down-regulated in HCC (A) miR-142-3p expression in matched non-tumor (NT) and HCC (*n* = 43) as determined by qPCR. The boxes contain the values between the 25^th^ and 75^th^ percentiles, the lines across the boxes indicate the median, and the whiskers extend to the highest values excluding outliers and extremes. (B) Correlation of miR-142-3p expression with HCC patient's cumulative disease-free survival rates, as determined by log-rank test.

To determine whether miR-142-3p expression is clinically relevant in HCC, we then extended our studies to the use of clinical tissue specimens. Expression of miR-142-3p was significantly repressed in HCC clinical samples, as compared with adjacent non-tumor liver tissues (*n* = 43; *p* < 0.001; Figure 2A). Compared by log-rank test analysis, patients with low miR-142-3p expression in HCC displayed worse overall disease-free survival (estimated mean = 32.349 months) when compared to those patients with high miR-142-3p expression (estimated mean = 52.824 months) (p = 0.049; Figure 2B). miR-142-3p expression was not found to correlate with another clinicopathological feature (Supplementary Table 1); but is inversely correlated with CD133 expression in HCC where we and others have previously reported CD133 to be preferentially expressed in HCC, but is detected at only low or absent levels in non-tumor liver tissues [3].

### miR-142-3p overexpression inhibits the ability of CD133-expressing HCC cells to self-renew, initiate tumors, invade, migrate and resist chemotherapy

**Figure 3:**
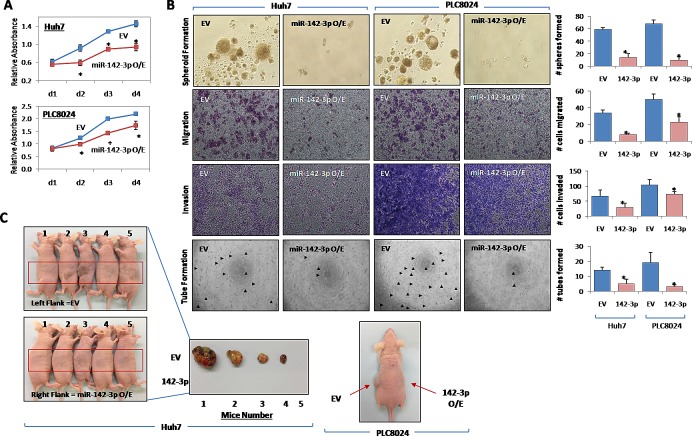
miR-142-3p regulates cancer stem cell-like properties in HCC (A) Growth curve of Huh7 and PLC8024 cells stably transduced with empty vector (EV) control or miR-142-3p, as determined by XTT cell proliferation assay. (B) Representative images of spheroid formation, migration, invasion and capillary tube formation assays in Huh7 and PLC8024 cells with miR-142-3p stably overexpressed as compared with their controls. (C) Representative image of the tumors formed in nude mice following injection of control cells (left flank) and miR-142-3p overexpressing (right flank) Huh7 and PLC8024 cells. Magnification at 400x.

**Figure 4:**
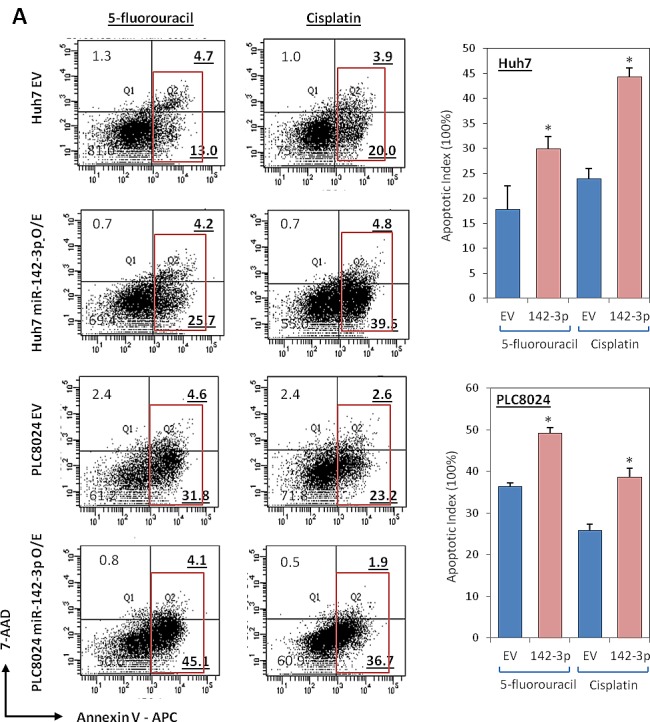
miR-142-3p overexpressing HCC cells exhibit a decreased ability to resist standard chemotherapy (A) AnnexinV-APC and 7-ADD flow cytometry analysis was performed to determine the extent of apoptosis in Huh7 and PLC8024 cells, with or without miR-142-3p overexpressed, following incubation with chemotherapeutic drugs cisplatin or 5-fluorouracil. Red box depicts both apoptotic and necrotic cells.

Since CD133 is a known functional liver CSC marker [4], we next examined whether miR-142-3p overexpression has any effect on inhibition of cancer and stem cell-like properties. Stable transduction with a lentiviral vector containing the primary transcripts of miR-142-3p produced high levels of mature miR-142-3p in Huh7 and PLC8024 HCC cells (Figure 1F). miR-142-3p transduced Huh7 and PLC8024 HCC cells showed diminished proliferation compared with control cells transduced with empty vector (EV) alone, as measured by XTT cell proliferation assay (Figure 3A). We also examined the effect of increased miR-142-3p expression on self-renewal and tumor growth. HCC cells with miR-142-3p overexpressed formed smaller and less spheroids than cells transduced with empty vector controls in a significantly shorter period of time (Figure 3B). Importantly, miR-142-3p transduced spheroids could not be passaged from one generation to another, whereas the untransduced spheres could, demonstrating their diminished self-renewal ability *in vitro*. Further, stable overexpression of miR-142-3p also led to a significant decrease in the ability of HCC cells to migrate and invade through an extracellular matrix coating (Figure 3B). Human umbilical vein endothelial cells (HUVEC) treated with conditioned media collected from miR-142-3p overexpressed showed a diminished ability to induce capillary tube formation as compared with medium collected from control cells (Figure 3B). When implanted into the flanks of immunodeficient mice, the growth of HCC cells with miR-142-3p overexpressed was significantly diminished. Of the 5 mice per group in Huh7 and PLC8024, empty vector control cells gave rise to tumors in 4 animals at approximately 5 weeks post-injection, while none of the animals injected with miR-142-3p overexpressing cells could form tumors (Figure 3C). In addition, HCC cells with miR-142-3p overexpressed were also more sensitive to chemotherapeutic reagents cisplatin and 5-fluorouracil, as evident by the significant increase in apoptotic / necrotic cells. Cisplatin and 5-fluorouracil-induced apoptosis in Huh7 HCC cells increased from 23.9% to 44.3% and 17.7% to 29.9%, respectively (Figure 4A top). Similar observations were likewise observed in another HCC cell line model PLC8024 (cisplatin – from 25.8% to 38.6% and 5-fluouracil – from 36.4% to 49.2%) (Figure 4A bottom).

### miR-142-3p regulates cancer stem cell-like properties in HCC via the direct targeting of CD133

**Figure 5:**
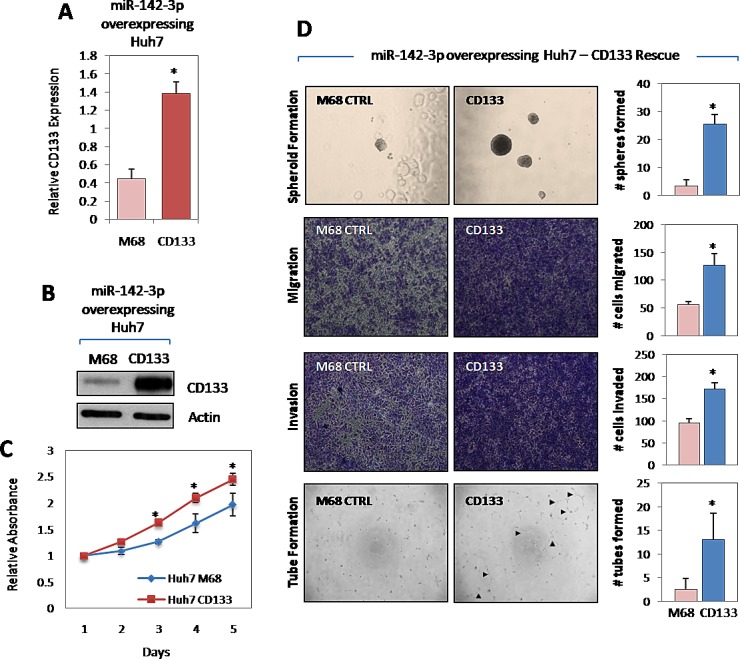
miR-142-3p regulates cancer stem cell-like properties in HCC via the direct targeting of CD133 (A-B) Stable overexpression of CD133 in Huh7 cells with miR-142-3p stably overexpressed was confirmed at both genomic and proteomic levels by qPCR and Western blot, respectively. M68 stands for pReceiver-M68 negative control vector in which CD133 was cloned into. (C) Growth curve of miR-142-3p overexpressing Huh7 cells stably transfected with M68 control vector and CD133, as determined by XTT cell proliferation assay. (D) Representative images of spheroid formation, migration, invasion and capillary tube formation assays demonstrating the ability of CD133 overexpression in miR-142-3p expressing Huh7 to rescue cancer and stem cell-like properties in HCC. Magnification at 400x.

To address whether the above observed phenotype is indeed due to the suppression of CD133 and not from the targeting of other cellular genes by miR-142-3p, a rescue experiment was performed. We co-transduced Huh7 HCC cells with miR-142-3p and CD133 or with miR-142-3p and vector control. Stable CD133 overexpression was confirmed at genomic and proteomic levels by qPCR and Western blot (Figure 5A-B). Indeed, Huh7 cells that have dual stable overexpression of miR-142-3p and CD133 had a compensated ability to proliferate, self-renew, migrate, invade and induce capillary tube formation in HUVECs (Figure 5C-D), as compared to Huh7 cells with miR-142-3p and vector control overexpressed.

## DISCUSSION

The CD133 epitope has now been identified as a tumor marker in a variety of cancer types for the purification of a specific subset of cells that demonstrate cancer stem cell phenotype. CD133 cancer stem cell subpopulations have been found in glioblastoma, colorectal cancer, lung cancer, head and neck cancer and hepatocellular carcinoma. Expression of CD133 has not only been linked to a more aggressive cellular behavior, it also been extensively correlated with advanced disease stage and worst overall survival. Our group has a long-standing interest in CD133 liver CSC research. We have previously identified a group of CSCs from HCC that is marked by their CD133 surface phenotype and bearing unique features that include the ability to self-renew, differentiate, initiate tumors *in vivo* and resist standard chemotherapy [1-3]. Further to its role as a liver CSC marker, CD133 also plays a functional role in regulating tumorigenesis of liver CSCs as evident via CD133 shRNA knockdown experiments [4]. CD133 represented only a minority of the tumor population in human HCC specimens (range ~1 to 25% by flow cytometry). Its expression had prognostic value in HCC as its presence was associated with advanced tumor stage as well as worse overall survival and higher recurrence rates [3]. In contrast, CD133 expression in their corresponding non-tumor liver tissues was significantly lower, if not at times almost absent [1, 3]. Recently, we also furthered our work to delineate the molecular mechanism by which CD133^+^ liver CSCs drive HCC, and found CD133 to confer resistance to standard chemotherapy through an activated Akt and Bcl-2 survival pathway [2]; and to promote angiogenesis through a deregulated neurotensin / IL-8 signaling pathway [4]. In addition, we also found HCC cells marked by the CD133^+^CD24^+^ phenotype to distinctly represent a metastatic liver CSC subset [15]. Importantly, our results are consistent with past studies by other groups where liver CSCs from HCC cell lines are also found to be marked by CD133 [5-8] and that CD133 expression is an important risk factor for overall survival in HCC [9], thus further demonstrating the prominence of CD133 in the cancer. These results suggest that CD133 liver CSCs represent an important subset of cells for HCC formation and recurrence. Two recent reports have shown elevated expression of CD133 in HCC to be associated with line-1 demethylation [16] and to be controlled by TGFβ1 [17]. Despite our growing understanding of this subset of cells, the mechanism by which CD133 in HCC is regulated remains limited. Critical to our understanding of CD133 liver cancer stem cells at a broader perspective, the present study is, to our knowledge, the first to identify miRNA epigenetic regulation on the control of CD133 expression and function in HCC. Analysis by in silico prediction and luciferase reporter assays identified miR-142-3p to play a critical role in the targeting of CD133. Expression of CD133 and miR-142-3p were inversely correlated in liver cell lines and HCC clinical samples. An inverse pattern of the two was also observed following lentiviral overexpression of miR-142-3p in CD133 expressing HCC cells Huh7 and PLC8024. Functional studies *in vitro* and *in vivo* found miR-142-3p to regulate the ability of HCC cells to confer cancer and stem cell-like properties, via the direct targeting of CD133. miR-142-3p has previously been implicated to control migration and invasion in HCC through negatively regulating RAC1 [18]. Further, shortly prior to the submission of this article, a study by Shen et al. found miR-142-3p to function as a tumor suppressor in colon cancer cells by targeting CD133, ABCG2 and LGR5 [19], though no rescue functional experiments were performed to delineate which of the three cellular targets was most crucial in mediating the effects of miR-142-3p in colon cancer cells. To address whether the deregulated cancer and stem cell-like phenotype is indeed due to the suppression of CD133 and not from the targeting of other cellular genes by miR-142-3p, rescue experiments were performed in our present study. In summary, our results show that the expression and function of the liver CSC marker CD133 is epigenetically regulated by miR-142-3p in HCC. Clinically, lower expression of miR-142-3p in HCC is significantly correlated with a worst overall survival, thus suggesting that miR-142-3p could possibly represent a good prognostic marker for the disease.

Lastly, we would also like to discuss an alternative point of view on therapeutic failures and the concept of “cancer stemloids” as introduced by Dr. Mikhail Blagosklonny [22, 23]. There is now solid data to show that cancer cells with stem cell-like properties represent a critical root of tumor growth. However, whether active proliferation is a defining functional property of this subset of cancer cells remains controversial [24]. Recently, it has been suggested that the selective killing of cancer stemloids (cancer cells with both stem cell-like and proliferating properties), possibly by cycle-dependent therapy, should be targeted for more efficient eradication of cancer. Regardless of the terminology used, we believe that successful irradiation of tumor growth would be best achieved through the use of a combination of therapy that targets both actively proliferating cancer cells as well as cancer cells with stem cell-like features and possibly together with drugs that target the tumor microenvironment.

## MATERIALS AND METHODS

### Cell culture

HepG2, Hep3B and SNU182 were obtained from the American Type Culture Collection (Manassas, VA). QSG-7701, QGY-7703, BEL7402 and PLC8024 were obtained from the Institute of Virology, Chinese Academy of Medical Sciences, Beijing, China. MiHA was provided by Dr. J.R. Chowdhury, Albert Einstein College of Medicine, New York [20]. Huh7 was provided by Dr. H. Nakabayashi, Hokkaido University School of Medicine, Japan [21]. All cell lines, except HepG2, were cultured in DMEM medium (Invitrogen, Carlsbad, CA) supplemented with 10% fetal bovine serum (Invitrogen), penicillin (500 U/ml) and streptomycin (500 μg/ml) in a 5% CO_2_ incubator at 37ΰC. HepG2 was maintained in EMEM medium (ATCC, Manassas, VA) supplemented with same concentration of FBS and penicillin and streptomycin. All cell lines used in this study were regularly authenticated by morphological observation and tested for absence of Mycoplasma contamination using MycoAlert kit (Lonza, Switzerland).

### Collection of non-tumor and HCC tissue clinical samples

A total of 43 matched HCC and adjacent non-tumor clinical samples were collected from the Sun Yat-Sen University Cancer Centre (Guangzhou, China). All samples used in this study were approved by the committee for ethical review of research involving human subjects at the Sun Yat-Sen University.

### RNA isolation, cDNA synthesis, RT-PCR and quantitative real-time PCR (qPCR)

Total RNA was isolated by Trizol reagent (Invitrogen). cDNA was synthesized using the PrimeScript RT Master Mix (Takara, Japan) and used for qPCR analysis. Sequences of primers used for CD133 amplification are as follow: forward 5'- TGGATGCAGAACTTGACAACGT – 3' and reverse 5'-ATACCTGCTACGACAGTCGTGGT-3'. β-actin was amplified as an internal control. For each qPCR reaction, equal amounts of cDNA were mixed with Power SYBR Green PCR master mix (Applied Biosystems, Carlsbad, CA) and 5 pmol each of forward and reverse primers. miRCURY LNA miRNA assays were used to quantify the expression levels of mature miR-142-3p (Exiqon). Total RNA was reverse transcribed by the Universal cDNA Synthesis Kit II (Exiqon), while quantification of mature miRNAs was performed using the ExiLENT SYBR Green Master Mix (Exiqon). SNORD48 small nuclear RNA was amplified as an internal control. qPCR for both mRNA and miRNA detection was conducted at 95°C for 10 min, followed by 40 cycles of 95°C for 15 sec and 60°C for 1 min. Specificity was verified by melt curve analysis. The crossing threshold value was noted for each transcript and normalized to the internal control. The relative quantification of each mRNA and miRNA was performed using the comparative Ct method. Experiments were performed using an ABI Prism 7900 System and data were processed using ABI SDS v2.1 software (Applied Biosystems).

### Western blot

Quantified protein lysates were resolved on SDS-PAGE, transferred onto PVDF membrane (Millipore, Billerica, MA) and probed with mouse anti-human CD133/1 (W6B3C1, Miltenyi Biotec) or mouse anti-human actin (Santa Cruz, Dallas, TX), followed by incubation with secondary HRP-conjugated antibodies. Blots were visualized by chemiluminescence (Amersham, UK).

### Flow cytometry

Flow cytometry for CD133 expression was performed on liver cell lines using PE-conjugated monoclonal mouse anti-human CD133/1 (AC133, Miltenyi Biotec). Isotype control mouse IgG1-PE (eBioscience, San Diego, CA) served as a control. Samples were analyzed on a BD FACSCalibur (BD Biosciences, Franklin Lakes, NJ) and data were analyzed using CellQuest software (BD Biosciences).

### miRNA-mRNA target prediction

To identify potential binding miRNA partners for CD133, a search in the publicly available alogrithims TargetScan (www.targetscan.org) Release 6.2 (June 2012) and miRanda (www.microrna.org) August 2010 Release was performed.

### Luciferase reporter assay

Full-length wild-type CD133 3'UTR was amplified and cloned into the *MluI* and *HindIII* sites of a *firefly* luciferase (*f-luc*) reporter gene of a pMIR-REPORT vector (Ambion, Carlsbad, CA) in sense or antisense directions. All PCR products cloned into the plasmid were verified by DNA sequencing to ensure that they were free of mutations and in the correct cloning direction. Sequence of primers used for luciferase reporter assays are provided in Supplementary Table 2. 293 cells were co-transfected with 600 ng of either the sense or antisense *firefly* luciferase constructs, 0.1 μg of pRL-TK (Promega, Madison, WI) and 30 nM synthetic miR-142-3p molecules or mock control (Negative Control #1, non-targeting RNA oligonucleotide; Ambion). The pRL-TK *Renilla* luciferase plasmid was used as an internal control to correct for differences in both transfection and harvest efficiencies. Forty-eight hrs after transfection, *firefly* and *Renilla* luciferase activities were measured using the Dual-Luciferase Reporter Assay (Promega). Results were expressed as relative luciferase activity (*firefly* luciferase/*Renilla* luciferase).

### Lentiviral transduction and transfection

Lentiviral constructs (Lenti-miR^™^ microRNA precursor clones, System Biosciences, Mountain View, CA) expressing miR-142-3p or their respective empty vector (EV) controls (scramble control hairpin in pCDH-CMV-MCS-EF1-copGFP, System Biosciences) were packaged using the pPACKH1 Lentivector Packaging System (System Biosciences) and were used to infect Huh7 and PLC8024 HCC cells to establish cells constitutively expressing miR-142-3p. The transduction efficiency, as evaluated by GFP expression, was >90%. Stable clones were selected using puromycin. Cells were infected with lentiviral media at a multiplicity of infection of 10 in the presence of 8 mg/ml polybrene (Sigma-Aldrich) overnight in a 37ΰC incubator. For stable overexpression of CD133, negative control vector in pReceiver-M68 or CD133 in pReceiver-M68 (GeneCopoeia, Rockville, MD) was transfected into Huh7 cells using Lipofectamine 2000 Reagent (Invitrogen) with stable clones selected using puromycin.

### Foci formation assay

Proliferation rates were determined by colorimetric assay using crystal violet (Sigma-Aldrich), a cytochemical stain that binds to chromatin.

### Spheroid formation assay

Single cells were cultured in 300 μl of serum-free DMEM/F12 medium (Invitrogen) supplemented with 20 ng/ml human recombinant epidermal growth factor (Sigma-Aldrich), 10 ng/ml human recombinant basic fibroblast growth factor (Invitrogen), 4 μg/ml insulin (Sigma-Aldrich), B27 (1:50; Invitrogen), 500 U/ml penicillin (Invitrogen) and 500 μg/ml streptomycin (Invitrogen). Cells were cultured in suspension in poly-HEMA-coated 24-well plates. Cells were replenished with 30 μl of supplemented medium every second day. To propagate spheres *in vitro*, spheres were collected by gentle centrifugation and dissociated to single cells using TrypLE Express (Invitrogen). Following dissociation, trypsin inhibitor (Invitrogen) was used to neutralize the reaction, and the cells were cultured to generate the next generation of spheres.

### Chemoresistance assay

Cells were treated with various concentrations of 5-fluorouracil (5-FU) and cisplatin (Huh7 with 250 μg/mL 5-FU or 10 μg/mL cisplatin and PLC8024 with 250 μg/mL 5-FU or 5 μg/mL cisplatin) for 48 hrs; and then harvested and stained in binding buffer, 7-AAD and APC-conjugated Annexin-V as provided by the Annexin-V APC Apoptosis Detection Kit (BD Biosciences) according to manufacturer's instructions. Analysis was determined by FACSCanto II and FACSDiva software (BD Biosciences).

### Cell invasion and motility assay

Invasion and migration assays were conducted in 24-well BioCoat Matrigel Invasion Chambers (BD Biosciences) or 24-well Millicell hanging inserts (Millipore). Cells re-suspended in serum free DMEM were added to the top chamber and DMEM supplemented with 10% FBS was added to the bottom chamber as a chemoattractant. After 48 hrs incubation at 37°C, the number of cells that invaded through the Matrigel (invasion) or membrane (migration) was counted in 10 fields under 4x objective lens and imaged using SPOT imaging software (Nikon, Japan).

### Capillary tube formation assay

Huh7 and PLC8024 cells infected with miR-142-3p or empty vector controls were plated into 6-well plates in DMEM medium containing 10% FBS. Culture medium was replaced by fresh medium without FBS after 24 hrs. Cell supernatants were collected and filtered after incubation for a further 24 hrs. HUVECs (1x10^4^) were seeded into 96-well plates and then treated with the tumor cell supernatant for 48 hrs. Capillary tube formation assays were then conducted using BD Matrigel Basement Membrane Matrix (BD Biosciences), according to the manufacturer's instructions.

### Animal experimentations

The study protocol was approved by and performed in accordance with the Committee of the Use of Live Animals in Teaching and Research at The University of Hong Kong. Tumorigenicity was determined by subcutaneous injection into the flank of 4-to-5 week old nude mice. Specifically, HCC cells infected with miR-142-3p or empty vector controls were injected subcutaneously in complete medium. Each group contained five animals. Cryosections (5 μm thick) were stained with hematoxylin and eosin stain. Animals that were injected with tumor cells but showed no sign of tumor burden were generally terminated three months after tumor cell inoculation, and animals were opened up at the injection sites to confirm that there was no tumor development.

### Statistical analyses

All statistical analyses were performed using PASW Statistics 18.0 (SPSS Inc., Chicago, IL), with the exception of the significance in bar graphs, in which case analyses were performed by applying the independent *t*-test using Microsoft Office Excel software (Microsoft Corp., Redmond, WA). A *p*-value of less than 0.05 was considered significant. miR-142-3p levels in HCC and adjacent non-tumor tissues were compared by paired Student *t* test. Differences in miR-142-3p expression among various clinicopathological stages were analyzed by Pearson chi-square (χ2) test or Fisher's Exact test, where appropriate. Cases with ΔCt lower than the mean value were classified as having high miR-142-3p expression, while cases with ΔCt higher than the mean value were classified as having low miR-142-3p expression. The Kaplan-Meier method and the log-rank test were used to compare the survival, defined as the time from surgery until death (living patients were censored at the time of their last follow up).

## SUPPLEMENTARY INFORMATION AND TABLES


