# Supplementary material for: Regulatory role of miR-142-3p on the functional hepatic cancer stem cell marker CD133
Source: Oncotarget. 2014 Jul 5;5(14):5725–35. doi: 10.18632/oncotarget.2167 (PMC4170635; doi:10.18632/oncotarget.2167)
Supplement: Supplementary file 1 [file oncotarget-05-5725-s001.pdf]

Regulatory role of miR-142-3p on the functional hepatic cancer stem cell marker CD133

Supplementary Information

Supplementary Table 1: Clinicopathological correlation of miR-142-3p expression in HCC.

Variable	*n*	HCC		*p* value
		Low miR-142-3p *n* (%)	High miR-142-3p *n* (%)	
**Gender**				
Male	34	18 (44.4)	16 (55.6)	
Female	9	4 (52.9)	5 (47.1)	0.721
**Age**				
≥50	24	11 (57.9)	13 (42.1)	
<50	19	11 (45.8)	8 (54.2)	0.432
**TNM Stage**				
I and II	30	16 (53.3)	14 (46.7)	
III (A, B, C)	13	6 (46.2)	7 (53.8)	0.665
**Tumor Number**				
1	32	16 (50)	16 (50)	
≥2	11	6 (54.5)	5 (45.5)	0.795
**Tumor Size** [2]				
<5	13	7 (53.8)	6 (46.2)	
≥5	30	15 (50)	15 (50)	0.817
**HBV Surface Antigen** [1]				
Positive	34	18 (52.9)	16 (47.1)	
Negative	8	4 (50)	4 (50)	1.000
**Differentiation** [1]				
Well	3	0 (0)	3 (100)	
Moderate	20	11 (55)	9 (45)	
Poor	19	11 (57.9)	8 (42.1)	0.256
**Adjacent Organ Invasion**				
Positive	6	1 (16.7)	5 (83.3)	
Negative	37	21 (56.8)	16 (43.2)	0.095
**Vascular Invasion**				
Yes	8	3 (37.5)	5 (62.5)	
No	35	19 (54.3)	16 (45.7)	0.457
**Cirrhosis** [1]				
No	11	6 (54.5)	5 (45.5)	
Mild	17	9 (52.9)	8 (47.1)	
Moderate	12	6 (50)	6 (50)	
Severe	2	1 (50)	1 (50)	1.000
**Capsule**				
Complete	12	7 (58.3)	5 (41.7)	
Incomplete	14	6 (42.9)	8 (57.1)	
No	17	9 (52.9)	8 (47.1)	0.721

[1] Partial data not available. Statistics based on available data only.

[2] Measured by the length of the largest tumor nodule.

Supplementary Table 2: Primer sequences for luciferase reporter assays.

	**Forward Primer (5' – 3')**	**Reverse Primer (5' – 3')**
3'UTR CD133 sense	ATACGCGTAAGGCAACGGCACCA	CGAAGCTTTGTAAACTGGTGTGTGT
3'UTR CD133 antisense	GGAAGCTTAAGGCAACGGCACCA	AAACGCGTTGTAAACTGGTGTGTGT
